# α-Cyclodextrin and α-Cyclodextrin Polymers as Oxygen Nanocarriers to Limit Hypoxia/Reoxygenation Injury: Implications from an In Vitro Model

**DOI:** 10.3390/polym10020211

**Published:** 2018-02-22

**Authors:** Saveria Femminò, Claudia Penna, Federica Bessone, Fabrizio Caldera, Nilesh Dhakar, Daniele Cau, Pasquale Pagliaro, Roberta Cavalli, Francesco Trotta

**Affiliations:** 1 Department of Clinical and Biological Sciences, University of Turin, 10043 Orbassano, Italy; saveria.femmino@unito.it (S.F.); claudia.penna@unito.it (C.P.); daniele.cau@edu.unito.it (D.C.); pasquale.pagliaro@unito.it (P.P.); 2 Department of Drug Science and Technology, University of Turin, 10125 Turin, Italy; f.bessone@unito.it (F.B.); roberta.cavalli@unito.it (R.C.); 3 Department of Chemistry, University of Turin, 10125 Turin, Italy; fabrizio.caldera@unito.it (F.C.); dhakar.neelesh89@gmail.com (N.D.)

**Keywords:** α-cyclodextrin, α-cyclodextrin nanosponges, α-cyclodextrin polymers, oxygen delivery, myocardial infarction, ischemia, reperfusion

## Abstract

The incidence of heart failure (HF) is increasing worldwide and myocardial infarction (MI), which follows ischemia and reperfusion (I/R), is often at the basis of HF development. Nanocarriers are interesting particles for their potential application in cardiovascular disease. Impaired drug delivery in ischemic disease is challenging. Cyclodextrin nanosponges (NS) can be considered innovative tools for improving oxygen delivery in a controlled manner. This study has developed new α-cyclodextrin-based formulations as oxygen nanocarriers such as native α-cyclodextrin (α-CD), branched α-cyclodextrin polymer (α-CD POLY), and α-cyclodextrin nanosponges (α-CD NS). The three different α-CD-based formulations were tested at 0.2, 2, and 20 µg/mL to ascertain their capability to reduce cell mortality during hypoxia and reoxygenation (H/R) in vitro protocols. H9c2, a cardiomyoblast cell line, was exposed to normoxia (20% oxygen) or hypoxia (5% CO_2_ and 95% N_2_). The different formulations, applied before hypoxia, induced a significant reduction in cell mortality (in a range of 15% to 30%) when compared to samples devoid of oxygen. Moreover, their application at the beginning of reoxygenation induced a considerable reduction in cell death (12% to 20%). α-CD NS showed a marked efficacy in controlled oxygenation, which suggests an interesting potential for future medical application of polymer systems for MI treatment.

## 1. Introduction

Advanced and smart polymer-based nanocarriers were designed for tissue-specific targeted delivery, triggered release, and co-delivery of synergistic drug combinations to develop safer and more efficient therapeutics. The heart is a metabolically demanding organ and mismatches between supply and demand of oxygen may occur, which could lead to hypoxia and cardiac cell distress. Hypoxia is a pathological condition for many diseases and it particularly occurs in acute coronary occlusion, which leads to cardiac cell death. This is the first phase of acute myocardial infarction (AMI). Hypertrophic surviving cells and heart failure are consequences of AMI. These serious outcomes of coronary occlusion can be limited by early and beneficial reperfusion. Full reperfusion is usually performed in primary angioplasty, but it may be deleterious (reperfusion injury) [1]. Actually, reperfusion/reoxygenation could be a hazardous practice. During ischemia, oxygen shortage may reduce oxidative phosphorylation and lead to a drop in intracellular pH as well as mitochondrial membrane depolarization. However, reperfusion might induce the massive formation of radical oxygen species (ROS) and a cascade of deleterious events leading to cardiac death and the inflammatory process known as redox stress. Moreover, ROS may be involved in triggering cardioprotective pathways through redox signalling [2,3,4,5]. Therefore, the production of devices that allow controlled delivery of oxygen can be of paramount importance for avoiding stress and favoring redox signalling.

Indeed, in order to reduce injury associated with ischemia/reperfusion (I/R), several studies have proposed a series of cardio-protective maneuvers including the application of brief periods of ischemia before or after prolonged ischemia. Ischemic preconditioning or post-conditioning might reduce significant I/R damage [2,3,4,6,7]. While preconditioning procedures may be applied to certain programmed clinical conditions that can jeopardise the heart, treatment after ischemia (post-conditioning mode) is clinically feasible even in unpredictable conditions. Preconditioning and post-conditioning protection can be induced by several stimuli besides ischemia such hypoxia, “oxygen tension control”, and several endogenous and exogenous compounds [2,3,4,8] all of which may act via redox signaling. Since post-conditioning depends on redox signaling, I/R injury may be conditioned by the reoxygenation modality in early reperfusion [9,10]. Hence, oxygen tension and the oxygen delivery patterns may play a crucial role in determining I/R injury.

Slow re-flow procedures have been proposed as experimental protective strategies. Intermittent re-flow (post-conditioning mode) has gained much more attention because, compared to slow re-flow, it does not present the problem of stagnant blood and consequent white blood cell infiltration [2,11]. However, intermittent reperfusion has raised several concerns in the clinical scenario and full reperfusion is still the treatment of choice [12]. 

Several studies have examined the ability to target nanoparticles for drug delivery to regions affected by organ ischemia. However, to the best of our knowledge, no study has tested a polymer nanoparticle approach for delivering oxygen in a controlled manner to limit I/R injury. Since nanoparticle approaches for oxygen delivery are theoretically feasible, this study aimed at testing the protective potential of cyclodextrin-based nanoparticles that can deliver oxygen in a highly controlled manner. Cyclodextrin-based nanosponges are innovative nanosized polymer systems consisting of cross-linked cyclodextrins nanostructured within a three-dimensional network. 

Cyclodextrins are a class of cyclic glucopyranose oligomers obtained from starch by enzymatic action. Cyclodextrins have a characteristic toroidal shape that forms a truncated cone-shaped lipophilic cavity. The main common native cyclodextrins are named α, β, and γ which comprise six, seven, and eight glucopyranose units, respectively. Cyclodextrins may include compounds whose size and polarity are compatible with that of their cavity. Moreover, due to the lipophilicity of the internal cavities, cyclodextrins are able to extract cholesterol from cell membranes [13]. As a consequence, the cytotoxicity of cyclodextrins was often tested in vitro through their hemolytic activity. The ability to induce hemolysis depends on the types of the cyclodextrin ring.

Cyclodextrin-based nanosponges exhibited a marked capability of incorporating many types of molecules including small molecules, macromolecules, and gases [14].

Concerning the safety aspect, β-CD-based NS proved to be biocompatible, biodegradable, and had negligible toxicity in preclinical studies in various animal models [15,16,17]. We took into account α-cyclodextrin and its ability to synthesize two polymer derivatives. α-CD contains six glucopyranose units and consequently a smaller internal cavity. Its safety profile was deeply investigated as candidate for pharmaceutical applications [13,18]. Recently, its cytotoxicity was evaluated on murine microvascular endothelial cells and compared with modified cyclodextrins [19,20].

Cyclodextrin nanosponges (NS) are suitable for oxygen transport, which was previously shown [21]. The major advantage of nanosponges is that they allow to “plan” the oxygen delivery pattern. The main objective of this study is to verify whether oxygenated or non-oxygenated (nitrogen filled) NS given in pre- or post-conditioning mode at different concentrations can reduce cell mortality or whether it can exacerbate cell death in a hypoxia/reoxygenation (H/R) in vitro model in H9c2 cardiomyocytes.

## 2. Materials and Methods 

α-Cyclodextrin was kindly provided by Wacker Chemie AG (Munich, Germany) and desiccated in the oven at 80 °C for at least 24 h before use. All other chemicals employed for the synthesis of CD polymers were purchased from Sigma-Aldrich (Steinheim, Germany) and used as received.

### 2.1. Synthesis of Soluble α-CD Polymer

A soluble hyper-branched α-CD polymer (α-CD POLY) was prepared following the procedure previously reported by Trotta et al. [22] with minor changes. In detail, 0.977 g (1.00 mmol) of α-CD were solubilized in 6.00 mL of dimethyl sulfoxide (DMSO) while stirring at an ambient temperature in a 20 mL vial. Subsequently, 1.00 mL (7.17 mmol) of triethylamine and 2.629 g (12.05 mmol) of pyromellitic dianhydride (PMDA) were added. After 24 h, the polymerization reaction was quenched by pouring the viscous solution into an excess of ethyl acetate. After precipitation, the synthesised polymer was recovered by vacuum filtration, washed with an excess of ethyl acetate, and left to dry. Finally, the polymer was solubilized in deionized water (approximately 100 mL) and freeze-dried. The polymer was stored in a desiccator after lyophilization.

### 2.2. Synthesis of Insoluble α-CD Nanosponge

An insoluble hyper-cross-linked α-CD polymer named α-CD nanosponge (α-CD NS) was synthesized by dissolving 1.954 g (2.01 mmol) of α-CD in 8.00 mL of DMSO while stirring at ambient temperatures in a 20 mL vial. Then, 2.00 mL (14.35 mmol) of triethylamine and 1.752 g (8.03 mmol) of PMDA were added [23]. In a few minutes, the cross-linking reaction led to the formation of a rigid gel. During the next several days, the gel was ground in a mortar and washed with an excess of deionized water under vacuum filtration. Finally, the NS was rinsed twice with acetone and left to dry. Further purification was performed by Soxhlet extraction by extracting the α-CD NS in acetone for approximately 24 h to remove the unreacted reagents.

### 2.3. Preparation of α-CD-based Formulations

Three types of α-CD-based formulations were prepared as oxygen delivery systems. For this purpose, α-CD, α-CD POLY, and α-CD NS were weighed and suspended in a NaCl solution (0.9%, *w*/*v*) to obtain an α-CD solution at a concentration of 1.3 mg/mL. Both the α-CD POLY solution and the α-CD NS suspension had a concentration of 40 mg/mL. Since α-CD NS is insoluble, it was first homogenized with a high-shear mixer (ltra-Turrax^®^, Konigswinter, Germany) at 24,000 rpm for 20 min to obtain a nano-suspension that presented a uniform particle size. The three α-CD-based formulations were saturated with oxygen by using an oxygen purge with a gas concentration up to 35 mg/mL.

### 2.4. Characterization of α-CD-Based Formulations

All α-CD formulations were characterized by determining pH and tonicity. The surface morphology of α-CD-based polymers in powder was determined by scanning electron microscopy (SEM) using a Leica Stereoscan410 (Wetzlar, Germany). The samples were observed after Au metallization. The voltage used was in the 5 kV to 10 kV range.

α-CD NS formulation was in vitro characterized to evaluate the size and surface charge. The average diameter and polydispersity index of the formulation was measured by using photocorrelation spectroscopy (PCS) with a 90 Plus instrument (Brookhaven, NY, USA) at a fixed angle of 90° and a temperature of 25 °C after dilution with filtered water. Each value represents the average of five measurements of three different sample batches. The zeta potential was determined by using electrophoretic mobility with a 90Plus instrument (Brookhaven, NY, USA). The zeta potential determinations were performed at blood plasma ionic strength (0.15 M). For zeta potential determination, diluted samples of α-CD-based formulations were placed in an electrophoretic cell to which a rounded 15 V/cm electric field was applied. Each value reported is the average of 10 measurements of 3 different formulations.

### 2.5. Hemolytic Assay

To determine hemolytic activity, 100 microliters of α-CD-based formulations were incubated at 37 °C for 90 min with blood (1:4 *v*/*v*) obtained after suitable dilution with freshly prepared phosphate buffer saline (pH 7.4). After incubation, sample-containing blood was centrifuged at 1000 rpm for five minutes to separate plasma. The amount of hemoglobin released due to hemolysis was determined spectrophotometrically (absorbance readout at 543 nm using a DU^®^ 730, Beckman Coulter, (Brea, CA, USA). Hemolytic activity was calculated using 0.9% NaCl solution (negative control) as reference. However, complete hemolysis (positive control) was induced by the addition of ammonium sulphate (20% *w*/*v*).

### 2.6. In Vitro Oxygen Release Determination

In vitro oxygen release from the three different types of α-CD-based formulations was investigated using the dialysis bag technique. The donor phase consisted of 3 mL of different α-CD formulations placed in a dialysis bag (cellulose membrane with molecular weight 12,000 Da to 14,000 Da) that was hermetically sealed and placed in 45 mL of the receiving phase. The receiving phase consisted of 0.9% NaCl solution with oxygen concentration previously reduced using an N_2_ purge up to 0 mg/L in order to mimic hypoxic conditions. Then, oxygen release kinetics from α-CD formulations were monitored for 48 h using an oximeter (HQ40d model, Hach, Loveland, CO, USA) at 25 °C.

### 2.7. Cell Culture

**Figure 1:**
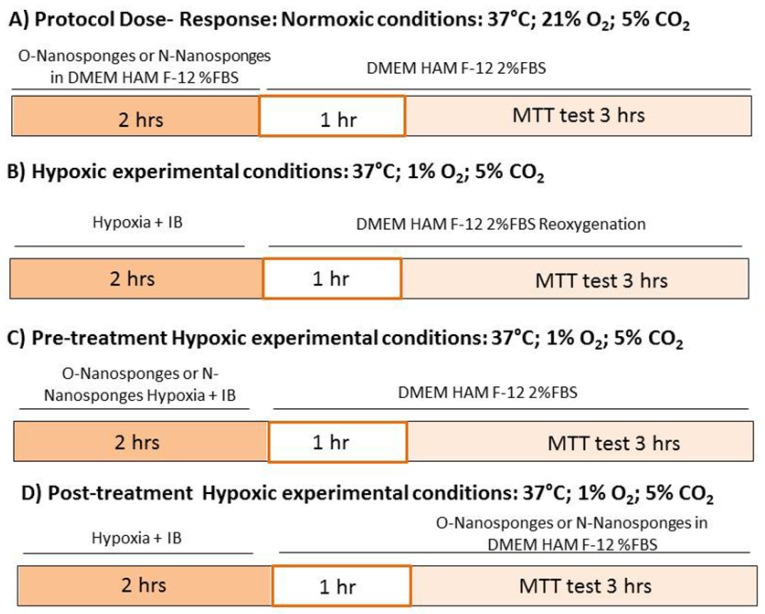
Experimental protocols and 3-(4,5-dimethylthiazol-2-yl)-2,5-diphenyltetrazolium bromide (MTT) test. IB: ischemic buffer. (**A**–**D**) are different experimental conditions.

Commercially available H9c2 samples were obtained from the American Type Culture Collection (ATCC; Manassas, VA, USA). H9c2 samples were grown in Dulbecco’s modified Eagle’s medium nutrient mixture F-12 HAM (DMEM) and supplemented with 10% fetal bovine serum (FBS) and 1% (*v*/*v*) streptomycin/penicillin (Wisent Inc., Quebec, QC, Canada) at 37 °C and 5% CO_2_ [24]. When the cells reached 80% confluence, they were removed from the flask, counted in the Burker chamber, and plated in a 96-well plate with 5000 cells/well density. After 48 h, the cells were used for dose–response analysis and hypoxia–reoxygenation (H/R) protocols (see Figure 1).

Upon completion of the protocols, the cells were washed with DMEM for three hours and an 3-(4,5-dimethylthiazol-2-yl)-2,5-diphenyltetrazolium bromide (MTT) assay was performed (see below).

### 2.8. Stability Determination of α-CD-based Formulations in Cell Culture Medium and Ischemic Buffer

The stability of α-CD-based formulations was determined in cell culture medium (DMEM HAM F12, 2% FBS), ischemic buffer, and NaCl 0.9% used as a negative control. α-CD-based formulations were incubated with three different solutions at a concentration of 100 μg/ml at 37 °C. Z-potential values were measured as described before over time (*t* = 0 h, 3 h, and 6 h). 

### 2.9. Protocols

#### 2.9.1. Normoxic Experimental Conditions (Dose–Response Studies)

To verify either oxygenated or non-oxygenated (Nitrogen) cell toxicity, α-CD-based formulations were tested at different concentrations (0.2 µg/mL, 2 µg/mL, and 20 µg/mL) (see Figure 1A).

Therefore, cell vitality of the Untreated Control Group (cells with DMEM HAM F12, 2% bovine serum only, CTRL) was compared against cell vitality of cells exposed to the following α-CD-based formulations, which are comprised of two types of α-CD nanosponges including either soluble (α-CD POLY) or insoluble (α-CD NS) and native α-CD in normoxic conditions (21% O_2_ and 5% CO_2_).

- Oxygenated α-CD POLY Groups (O-α-CD POLY) and Nitrogen α-CD POLY Groups (N-α-CD POLY)
- Oxygenated α-CD NS Groups (O-α-CD NS) and Nitrogen α-CD NS Groups (N-α-CD NS)
- Oxygenated α-CD Groups (O-α-CD) and Nitrogen α-CD Groups (N-α-CD)

#### 2.9.2. Hypoxia/Reoxygenation Experimental Conditions

To study the response to the hypoxia–reoxygenation (H/R) in vitro protocol, the cells were exposed to hypoxia (5% CO_2_ and 95% N_2_) for two hours and subsequently reoxygenated (21% O_2_ and 5% CO_2_) for one hour. In particular, during hypoxia, H9c2 cells were exposed to simulated ischemia by replacing the medium with an “ischemic buffer”. This buffer contained 137 mM NaCl, 12 mM KCl, 0.49 mM MgCl_2_, 0.9 mM CaCl_2_ 2H_2_O, 4 mM HEPES, and 20 mM sodium lactate (pH 6.2) [25]. At the end of the hypoxic period, the cells were subjected to reoxygenation with a new medium (DMEM HAM F-12 2%FBS) for 1 h. At the end of reoxygenation, the cell vitality was assessed using the MTT test (see Figure 1B).

#### 2.9.3. Pre-Treatment with α-CD-Based Formulations

To mimic preconditioning, the cells were exposed to α-CD-based formulations that were either oxygenated or which contained nitrogen as described above and then subjected to the hypoxia–reoxygenation protocol, which is mentioned above (Figure 1C).

#### 2.9.4. Post-Treatment with α-CD-Based Formulations

To mimic post-conditioning, the oxygenated or nitrogen NSs were added to the cells at the beginning of reoxygenation (see Figure 1D).

### 2.10. MTT Assay

When all experiments were completed, the cell vitality was assessed using the 3-(4,5-Dimethylthiazol-2-yl)-2,5-diphenyltetrazolium bromide (MTT) kit. MTT (10 µL/well, Sigma, St. Louis, MO, USA) was added to each well. Cells were incubated for another three hours at 37 °C. Then 100 µL of dimethyl sulfoxide (DMSO, Sigma, St Louis, MO, USA) samples were added to each well and the plates were shaken for five minutes. Each experiment was performed three times. The plates were read by a spectrophotometer at 570 nm to obtain optical density values [26].

### 2.11. Statistical Analysis

All values were expressed as a mean ± SEM and were analyzed using the Analysis of Variance (ANOVA) test followed by Bonferroni’s post-test and the *t*-test. A value in the range of *p* < 0.05 was considered statistically significant.

## 3. Results

### 3.1. Physicochemical Characterisation of α-CD-Based Formulations

The three α-CD-based formulations were prepared in NaCl (0.9% *w*/*v*), which are suitable for biological studies. The pH value of the three different α-CD-based formulations was 5.50 and tonicity was about 300 mOSM.

The α-CD POLY showed an average molecular weight of 26 KDa and a content of α-CD macrocycles corresponding to 27.10%. The aqueous solubility of α-CD and α-CD POLY is 145 mg/mL and more than 250 mg/mL while α-CD NS entities are insoluble.

The results of the physicochemical characterization of the α-CD NS formulation are stated in Table 1. 

**Figure 2:**
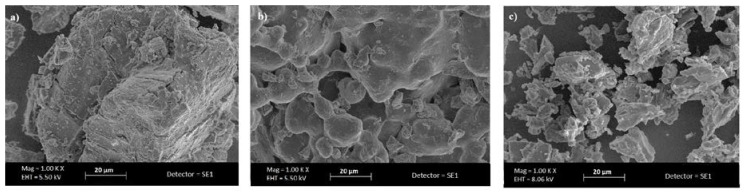
Scanning electron microscopy (SEM) images of (**a**) α-cyclodextrin (**b**) α-cyclodextrin polymer, and (**c**) α-cyclodextrin nanosponges (Magnification 1000×).

α-CD NS was about 850 nm in size and had a negative surface charge with a high value of zeta potential (about −36 mV) that was suitable for avoiding the nano-sponge aggregation phenomenon. PDI values lower than 0.2 indicate a nearly homogeneous particle size distribution. Figure 2 reports SEM images of α-CD-based polymers in comparison with α-CD.

The pictures underlined the different morphology of α-CD, α-CD POLY, and α-CD NS in powder form.

### 3.2. Evaluation of α-CD-Based Formulation Biocompatibility

**Figure 3:**
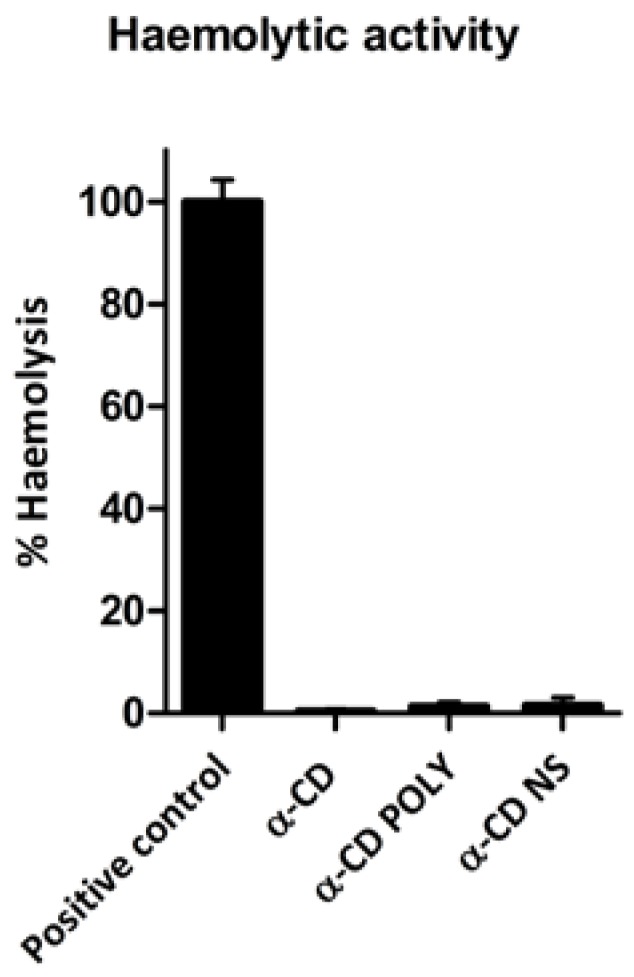
α-CD-based formulations presented negligible hemolytic activity. Results were expressed as percentage of total hemolysis (positive control), which was obtained when red blood cells were incubated with ammonium sulphate (20% *w*/*v*). Each bar represents the mean ± Standard Deviation (SD) of the three experiments.

Red blood cell (RBC) hemolysis was performed to assess the formulation’s biocompatibility. Negligible hemolysis was observed (see Figure 3) for α-CD-based formulations at the concentration used in the biological assays, which confirms their biocompatibility. These results are consistent with literature data, which report that α-CD exhibits hemolysis starting from 6 mM [27].

### 3.3. In Vitro Oxygen Release Kinetics

**Figure 4:**
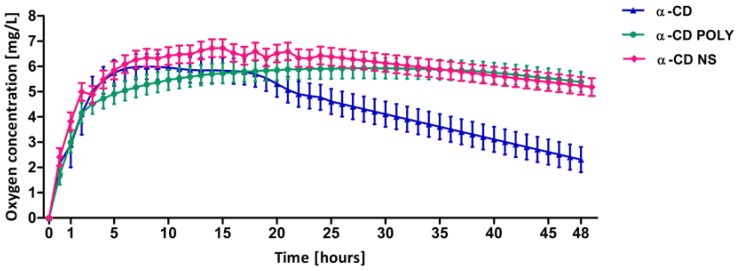
In vitro oxygen release from α-CD-based formulations over time measured by an oximeter. Each point represents the mean ± SD of the three experiments.

The in vitro oxygen release profile obtained from the three different α-CD-based formulations is noted in Figure 4. All oxygen-loaded α-CD-based formulations were able to store and slowly release oxygen with prolonged and constant release kinetics. The release profile has two phases for all the systems. α-CD showed a faster release than the other two formulations. This behavior underscores the role played by the presence of the polymer.

### 3.4. Stability Determination of α-CD-Based Formulations in Cell Culture Medium and Ischemic Buffer

**Figure 5:**
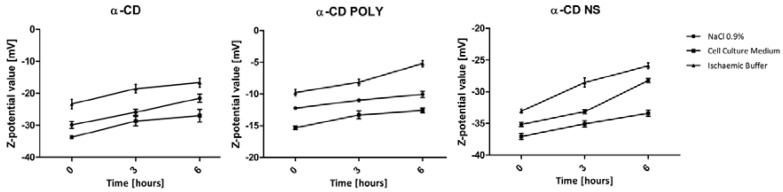
Zeta potential values of α-CD-based formulations in NaCl 0.9% (negative control) cell culture medium in an ischemic buffer over time. Each point represents the mean ± SD of three experiments.

The zeta potential behavior of the three formulations was monitored up to six hours in cell culture medium and ischemic buffer in order to confirm the system stability during the time range used in the following biological assays (see Figure 5).

No significant changes in zeta potential values were observed, which suggests that no aggregation phenomena occurred. Preliminary experiments and data reported in the literature [14] demonstrated the chemical stability of α-CD POLY and α-CD-NS over time. In addition, no changes of oxygen concentration were seen with regard to storing NS aqueous suspensions for 30 days in sealed vials [21].

### 3.5. Dose–Response in Normoxic Conditions

**Figure 6:**
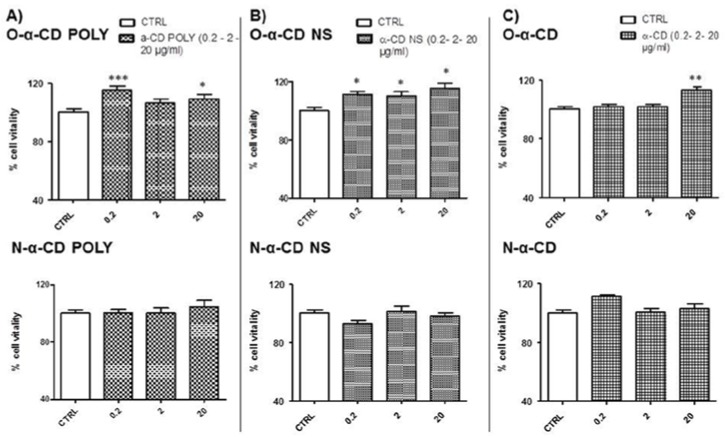
Dose–response in normoxic conditions. (**A**) Treatment with O-α-CD POLY (0.2, 2, 20 µg/mL) and N-α-CD POLY (0.2, 2, 20 µg/mL); (**B**) Treatment with O-α-CD NS (0.2, 2, 20 µg/mL) and N-α-CD NS (0.2, 2, 20 µg/mL); (**C**) Treatment with O-α-CD (0.2, 2, 20 µg/mL) and N-α-CD (0.2, 2, 20 µg/mL), compared to the untreated control group (CTRL). Data were normalized to the mean value in control conditions and expressed as a percentage.

Results obtained with different concentrations (0.2, 2, and 20 µg/mL) of oxygenated (upper panels) and non-oxygenated (nitrogen in lower panels) α-CD-based formulations in normoxic conditions are available in Figure 6. The exposure of cells to different types of nitrogen-loaded (flushed) α-CD-based formulations for two hours did not affect cell vitality in all tested concentrations. On the other hand, treatment with oxygenated samples had a slight proliferative effect depending on concentration and type of α-CD-based formulations. In particular, O-α-CD POLY induced a significant increase in cell vitality when compared to untreated cells (CTRL) at two different concentrations (0.2 and 20 µg/mL, *p* < 0.001 and *p* < 0.05 vs. CTRL, respectively) (see Figure 6A). O-α-CD NS induced a significant increase in vitality in all tested concentrations (0.2 µg/mL and 2 µg/mL *p* < 0.05, 20 µg/mL *p* < 0.001 vs. CTRL; Figure 6B). For O-α-CD, we observed a significant increase in vitality when the cells were treated with 20 µg/mL only (*p* < 0.01 vs. CTRL, see Figure 6C upper panel).

### 3.6. Untreated Cells: Normoxic and H/R Conditions

**Figure 7:**
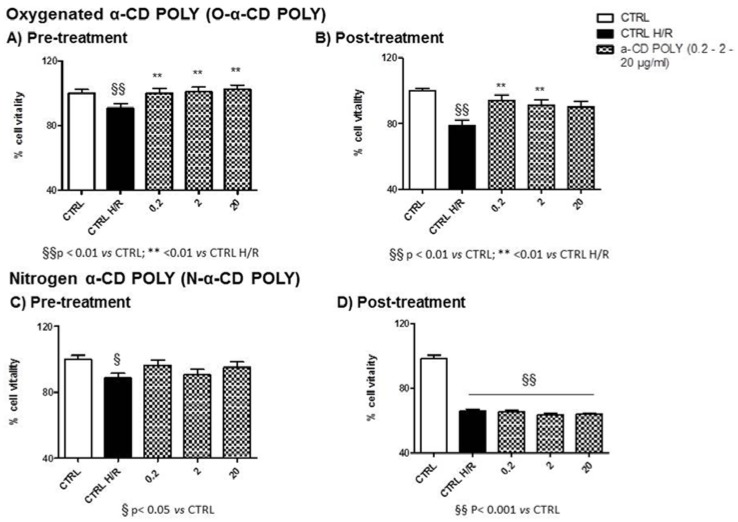
Cell vitality of α-CD POLY treated H9c2 cells and H/R conditions. (**A**) Pre-treatment with O-α-CD POLY (0.2, 2, and 20 µg/mL); (**B**) Post-treatment with O-α-CD POLY (0.2, 2, and 20 µg/mL); (**C**) Pre-treatment with N-α-CD POLY (0.2, 2, and 20 µg/mL); (**D**) Post-treatment with O-α-CD POLY (0.2, 2, and 20 µg/mL) compared to the untreated control group (CTRL) and the hypoxia–reoxygenation group (CTRL H/R). Data were normalized to mean value in control conditions and expressed as a percentage.

**Figure 8:**
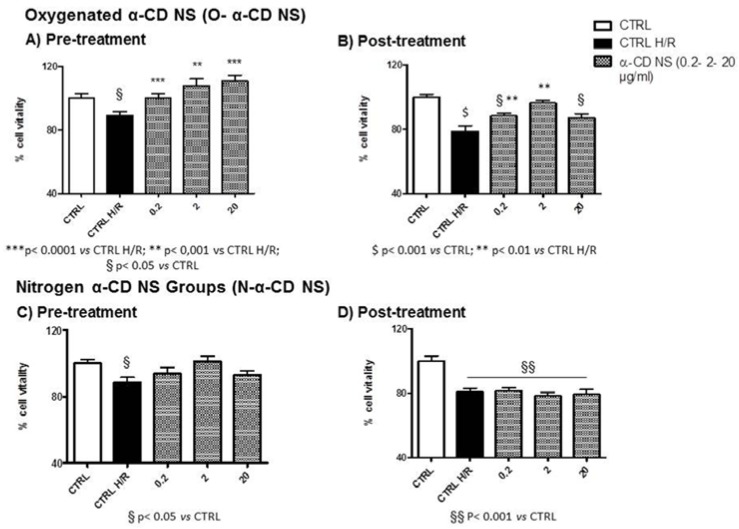
Cell vitality of α-CD NS treated H9c2 cells and H/R conditions. (**A**) Pre-treatment with O-α-CD NS (0.2, 2, and 20 µg/mL); (**B**) Post-treatment with O-α-CD NS (0.2, 2, and 20 µg/mL); (**C**) Pre-treatment with N-α-CD NS (0.2, 2, and 20 µg/mL); (**D**) Post-treatment with O-α-CD NS (0.2, 2, and 20 µg/mL) compared to the untreated control group (CTRL) and the hypoxia–reoxygenation group (CTRL H/R). Data are normalized to the mean value in control conditions and expressed as a percentage.

**Figure 9:**
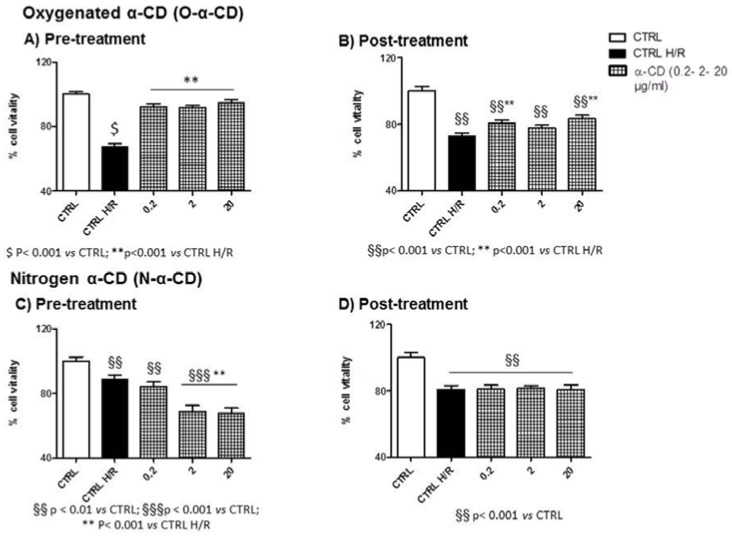
Cell vitality of α-CD treated H9c2 cells and H/R conditions. (**A**) Pre-treatment with O-α-CD (0.2, 2, and 20 µg/mL); (**B**) Post-treatment with O-α-CD (0.2, 2, and 20 µg/mL); (**C**) Pre-treatment with N-α-CD (0.2, 2, and 20 µg/mL); (**D**) Post-treatment with O-α-CD (0.2, 2, and 20 µg/mL), compared to the untreated control group (CTRL) and the hypoxia–reoxygenation group (CTRL H/R). Data were normalized to the mean value in control conditions and expressed as a percentage.

When comparing the first two bar graphs of all panels in Figure 7, Figure 8 and Figure 9, the untreated cells (not exposed to NS) at the end of the H/R protocol (CTRL H/R) displayed a 30% reduction in vitality (*p* < 0.05 CTRL vs. CTRL H/R in all cases).

### 3.7. NS-Treated Cells and H/R Conditions

#### 3.7.1. α-CD POLY

All concentrations of Oxygenated-α-CD POLY limited H/R damage when used during pre-treatment (see Figure 7A) or post-treatment (see Figure 7B). In all cases, vitality was significantly higher than in CTRL H/R conditions (Figure 7A,B). It must be mentioned that, in all cases (pre-and post-treatment), cell vitality resembled that of the normoxic CTRL.

Nitrogen-α-CD POLY were not protective when used during pre-treatment (see Figure 7C) or post-treatment (see Figure 7D). In all cases, vitality was not significantly higher than in CTLR H/R conditions (see Figure 7C,D). Moreover, in post-treated conditions, cells displayed reduced vitality when compared to normoxic conditions for all concentrations (CTLR; Figure 7D) whereas in pre-treatment conditions, cells only at 2 µg/mL H/R showed significantly reduced cell vitality when compared to the CTLR (*p* < 0.05, see Figure 7C).

#### 3.7.2. α-CD NS 

All concentrations of oxygenated-α-CD NS limited H/R damage when used during pre-treatment (see Figure 8A). However, when used during post-treatment (see Figure 8B), the vitality of cells exposed to a higher concentration of NS did not differ from the CTRL H/R. At all pre-treated concentrations, cells displayed a cell vitality similar to the normoxic CTRL (see Figure 6A). However, when used during post-treatment, the cells displayed vitality resembling the normoxic CTRL only for the 2 µg/mL concentration (Figure 8B).

Nitrogen-α-CD NS were not protective when used during pre-treatment (see Figure 8C) or post-treatment (see Figure 8D). In all cases, vitality was not significantly higher than in CTLR H/R conditions (see Figure 8C,D). In post-treated conditions, cells displayed reduced vitality compared to normoxic conditions for all concentrations (CTLR, see Figure 8D) whereas in pre-treatment conditions, no concentration significantly reduced cell vitality compared to the CTLR (see Figure 8C).

#### 3.7.3. α-CD

All concentrations of oxygenate-α-CD limited H/R damage when used during pre-treatment (see Figure 9A). However, when used during post-treatment (see Figure 9B), the vitality of cells exposed to an NS concentration of 2 µg/mL did not differ from the CTRL H/R. Moreover, in all pre-treated concentrations, cells displayed vitality resembling the normoxic CTRL (see Figure 9A). However, when used during post-treatment, no group displayed vitality resembling that of the normoxic CTRL (see Figure 9B). 

Nitrogen-α-CD particles were not protective when used during pre-treatment (see Figure 8C) or post-treatment (see Figure 9D). On the other hand, in pre-treated conditions, vitality was significantly higher than in CTLR H/R conditions for higher concentrations (see Figure 9C). In post-treated conditions, cells displayed reduced vitality when compared to normoxic conditions for all concentrations (CTLR, see Figure 9D). Cell vitality was significantly reduced when compared to the CTLR for all concentrations (see Figure 9C).

## 4. Discussion

Nanocarriers for oxygen delivery have been the focus of extensive research for the treatment of hypoxic tissues, infectious diseases, regenerative medicine, and wound healing [21,28,29,30,31,32,33].

In this study, two new α-CD-based polymers were synthesised as oxygen carriers. We had previously developed cyclodextrin-based nanosponges as oxygen carriers by cross-linking α, β, and γ-CD using carbonyldiimidazole [21]. α-CD nano-sponges showed the capability to store and release oxygen. Cyclodextrins can store gases in their cavity. Molecular encapsulation of gases in cyclodextrins was first studied by Cramer and Henglein [34]. Since gases have low molecular weight and a small size, α-CD was the most suitable compound for this application while, due to the higher dimension of the inner cavity, β-CD generally did not fit the requirements to host gases [35].

This paper investigates two new types of polymers obtained using α-CD and pyromellitic dianhydride (PMDA) as a cross-linker to obtain either a soluble polymer (hyperbranched) or an insoluble one (cross-linked) called a nanosponge (NS). The rationale for using α-CD is two-fold.

First, α-CD has a permitted daily exposure (PDE) of 0.2 mg/kg, which corresponds to 14 mg for a 70 kg human body. Based on the data, we can theorize that α-CD-based polymers could have an α-CD concentration that is suitable for future in vivo administration. Moreover, oxygen release kinetics might be slower since the α-CD cavity is smaller than those of other native cyclodextrins. α-CD cross-linked with PMDA were able to encapsulate, store, and release oxygen for a prolonged period of time. In addition, the polymer network may play a key role in oxygen storage and release. Treatment of myocardial infarction with nanotechnology has provided evidence of beneficial effects in both AMI and clinical studies.

This is a first-time study that evaluates different preparations of α-CD-based polymers incorporated with oxygen in a biological system and compared with native α-CD. These preparations provide clear protection against H/R-induced cell death when administered either before or after hypoxia. We also demonstrated that, at a different time of administration such as before or after the hypoxia protocol, these oxygen carriers induce protection. Our data suggest that in vitro oxygenated nanosponge treatment protects against reperfusion injury with a dose response. Considering a potential in vivo administration, the nanoparticle sizes are crucial parameters in the study. The dimensions significantly affect the pharmacokinetics and the bio-distribution of nano-delivery systems [36]. It is possible to reduce at nanoscale level a coarse material using milling processes. Top down methods such as high pressure homogenization can be exploited to reduce sizes of nanosponges and obtain an almost homogenous nanoparticle distribution, which was previously noted in [37,38]. Therefore, the size of α-CD-NS might be significantly decreased to obtain an aqueous nano-suspension suitable for future intravenous administration.

This study demonstrates a beneficial effect of controlled oxygen delivery with nano-sponges. Since these may be directly injected into the myocardial wall before starting full blood reperfusion, physicians may now possess a new tool for controlled oxygen delivery to limit ischemia/reperfusion injury.

Growing evidence supports the role of oxygenation levels and associated ROS generation in signal transduction pathways involved in heart conditioning [2,3,4,8]. Previous studies have demonstrated that a few minutes of both hypoxic and hyperoxic preconditioning might trigger beneficial ROS-dependent signaling, which may limit myocardial I/R injury. Similar beneficial ROS signaling has been described in the post-conditioning procedure with a few seconds of intermittent ischemia [2,9,10]. However, during the reperfusion phase, the effects of ROS are more complex and high levels of ROS may be detrimental [3]. Additionally, the dose–response curve we observed with oxygenated NS given during reoxygenation reveals a type of inverted U curve in which the middle dose (2 μg/mL) is the most effective and the higher dose (20 μg/mL) is not protective.

In conclusion, α-cyclodextrin and α-cyclodextrin polymers used as oxygen nanocarriers limit hypoxia–reoxygenation injury in a cardiac cell model both during pre-conditioning and post-conditioning. As such, these nanocarriers might be used to limit hypoxia–reoxygenation injury before a scheduled period of ischemia, which occurs during organ surgery, or during reperfusion treatment for infarction to provide controlled oxygenation. They possess interesting potential for future medical applications.

## Figures and Tables

**Table 1:** Physio-chemical characterization of an α-CD formulation. Each point represents the mean ± SD of the three different formulations (*n* = 3).

Formulation	Average diameter ± SD (nm)	PDI *	*Z*-potential ± SD (mV)
α-CD **	-	-	−29.12 ± 4.74
α-CD POLY **	-	-	−12.87 ± 4.60
α-CD NS	850.55 ± 57.90	0.17	−36.37 ± 3.58

* PDI = polydispersity index; ** Soluble in aqueous media.
